# The joint association of obesity and low handgrip strength with cognitive impairment and probable dementia: a cross-sectional study

**DOI:** 10.3389/fnut.2026.1813754

**Published:** 2026-07-17

**Authors:** Robinson Ramírez-Vélez, Miguel Germán Borda, Juan Carlos Calderón-González, Gonzalo Romero-Martínez, Mikel Izquierdo, Miguel A. Pérez-Sousa

**Affiliations:** 1 Navarrabiomed, IdiSNA, Hospital Universitario de Navarra (HUN), Universidad Pública de Navarra (UPNA), Pamplona, Spain; 2 CIBER of Frailty and Healthy Aging (CIBERFES), Instituto de Salud Carlos III, Madrid, Spain; 3 Facultad de Ciencias de la Educación, Unidad Central del Valle del Cauca (UCEVA), Tuluá, Colombia; 4 Department of Neurology, Clínica Universidad de Navarra, Pamplona, Spain; 5 Centre for Age-Related Medicine (SESAM), Stavanger University Hospital, Stavanger, Norway; 6 Department of Physical Education, Faculty of Education, University of Córdoba, Cordoba, Spain; 7 Epidemiology of Physical Activity and Fitness across Lifespan Research Group, University of Seville, Seville, Spain

**Keywords:** cognitive impairment, handgrip strength, Latin America, muscular strength and function, probable dementia

## Abstract

**Introduction:**

Obesity and low handgrip strength (HGS) have both been associated with cognitive impairment and dementia, but their combined association remains unclear. This study examined the association of obesity and low HGS, separately and jointly, with cognitive impairment and probable dementia in older Colombian adults.

**Methods:**

A secondary cross-sectional analysis was conducted using data from the Colombian Health, Well-Being, and Aging Survey (SABE Colombia 2015). The analytic sample included 4,656 community-dwelling adults aged ≥60 years. Obesity was defined as body mass index ≥30 kg/m^2^, and low HGS was defined using EWGSOP2 sex-specific cut-off points (<27 kg for men and <16 kg for women). Cognitive impairment was assessed using a revised version of the Mini-Mental State Examination. Probable dementia was defined as the co-occurrence of cognitive impairment and at least two limitations in instrumental activities of daily living.

**Results:**

Cognitive impairment was present in 12.2% of men and 14.2% of women, while probable dementia was observed in 4.9% of men and 6.6% of women. Low HGS, with or without obesity, was associated with higher odds of cognitive impairment in both sexes. For probable dementia, low HGS was associated with higher odds in both sexes, whereas obesity combined with low HGS was significantly associated with probable dementia only in women in the fully adjusted model. Obesity with normal HGS was not associated with either outcome.

**Discussion:**

Low HGS, rather than obesity alone, was associated with cognitive impairment and probable dementia in Colombian older adults. These findings support the relevance of considering muscular function when examining cognitive health in later life.

## 1 Introduction

Obesity and low handgrip strength are both associated with cognitive decline and dementia, but their combined effects are complex. Research indicates that while obesity in midlife is linked to impaired cognitive function, the relationship may change with age, potentially offering some protective effects against cognitive decline in older age (1). Conversely, low handgrip strength consistently predicts cognitive decline across various studies (2–4). Obesity is a leading cause of multiple chronic conditions and a major contributor to mortality and disability. Obesity in mid-life is associated with impaired cognitive function and increased risk of dementia, potentially due to metabolic consequences like type 2 diabetes. However, the protective effects of obesity in older age require further investigation. Obesity can develop early in life and persist through middle and old age (5).

In older adults, besides the amount of adipose tissue, the amount and quality of the muscle have shown to be a strong predictor of important outcomes, including mortality and cognitive decline (6). Several studies have indicated that in advanced age, higher BMI may correlate with specific protective effects, such as lower mortality and even lower rates of cognitive decline (7, 8). In this context, low HGS is a strong predictor of cognitive decline and dementia (9) Studies consistently show that individuals with weaker HGS experience more significant cognitive decline over time (10–12). Higher HGS is associated with a reduced risk of cognitive impairment, particularly in obese women. The mechanisms underlying this association remain uncertain. Nevertheless, potential contributors include the anti-inflammatory effects of exerkines, the production of myokines, and the roles of mitochondrial dysfunction and oxidative stress (13). The combination of obesity and HGS presents a nuanced picture. Overweight individuals with high HGS have lower odds of cognitive decline than those with normal weight and normal HGS strength. In obese women, muscular HGS significantly reduces the risk of cognitive impairment (2).

In Latin America, obesity is a significant public health issue, with some of the highest prevalence rates worldwide (14). As the region’s population ages rapidly, other usually concomitant related conditions such as dementia have come into focus (15). There are still significant gaps in understanding how increased adiposity and reduced muscular strength affect cognition. Therefore, this study aimed to investigate the associations of muscular strength (as measured by HGS) and obesity with cognitive impairment and probable dementia.

## 2 Methods

### 2.1 Study design and participants

Secondary data analysis was performed on a cross-sectional study using the Colombian Health, Well-Being and Aging Survey (SABE 2015, from the Spanish: SAlud, Bienestar and Envejecimiento). This study represents the first comprehensive examination of Colombia’s national population aged 60 years and older conducted from 2014 to 2015 by the Pan-American Health Organization and supported by the Epidemiological Office of the Ministry of Health and Social Protection of Colombia^1^ as described elsewhere (16). In addition, represents the biggest cross-sectional sample of older adults in Latin America. Participants for SABE Colombia were chosen through a multistage area probability sampling method, resulting in a total sample size of 23,694 individuals from 244 municipalities, encompassing both urban and rural areas. The institutional review boards involved in developing the SABE 2015 study (the University of Caldas, ID protocol CBCS-021-14, and the University of Valle, ID protocol 09–014 and O11-015) reviewed and approved the study protocol. Written informed consent was obtained from each individual before inclusion and completion of the first examination. One of the authors (RR-V.) applied to the Ministry of Health and Social Protection of Colombia and obtained permission to use publicly available data for research and teaching purposes (permission and details available at https://www.minsalud.gov.co/), in accordance with the Declaration of Helsinki (World Medical Association) and Resolution 8,430 from 1993, of the then Colombian Ministry of Health, on technical, scientific, and administrative standards for conducting research with humans (16). Additional methodological details are provided in Supplementary eFigure 1 in the Supplementary Material.

### 2.2 Data collection

The health survey included a medical history examination, an HGS examination, and questionnaires on health disorder history, lifestyle data, and anthropometric variables (16). Technical and medical staff performed physical tests following the standardized protocol for the SABE study (16). Height and body weight were measured using a portable stadiometer (SECA 213, Hamburg, Germany) and an electronic scale (Kendall graduated platform scale). Body mass index (BMI) was calculated as weight in kilograms divided by the square of height in meters, a standard method for determining obesity (16).

We used HGS as a measure of muscle strength and a proxy for sarcopenia in obese individuals (17). HGS was assessed in the SABE Study by a trained research nurse utilizing a Takei dynamometer (Takei Scientific Instruments Co., Tokyo, Japan). Before the assessment, the dynamometer was calibrated to ensure accurate and appropriate use. Participants were instructed to perform a grip assessment with the elbow joint fully extended and were encouraged to stand during the procedure if feasible. A practice trial was conducted to ensure that participants understood the protocol. HGS was measured thrice for each hand, alternating between hands in each trial, and the mean value was computed as the final score.

Cognitive impairment was assessed using the shortened Spanish-language version of the Mini-Mental State Examination (MMSE) applied in SABE Colombia, which ranges from 0 to 19 points. In accordance with the established cutoff for this instrument, a score of ≤12 was used to define cognitive impairment, whereas scores ≥13 indicated no cognitive impairment (18). Functional impairment was evaluated using four items from the Lawton and Brody instrumental activities of daily living (IADL) scale: telephone use, transportation, medication management, and financial management (19). Participants requiring help in two or more IADL were classified as functionally impaired. Probable dementia was operationally defined as the co-occurrence of cognitive impairment and functional impairment, consistent with previous population-based approaches used in Latin American epidemiological surveys (20). This definition does not represent a clinically confirmed diagnosis.

For the analysis of lifestyle characteristics, alcohol consumption was classified according to whether participants currently consumed alcohol, while cigarette smoking status was categorized according to whether participants currently smoked. A “proxy physical activity” assessment was conducted through three inquiries: (i) “Have you regularly exercised, including activities such as jogging or dancing, or engaged in vigorous physical activity at least three times per week over the past year?”; (ii) “Do you walk at least three times a week for a distance between nine and 20 blocks (approximately 1.6 km) without resting?”; (iii) “Do you walk at least three times a week for a distance of eight blocks (0.5 km) without resting?” Participants were classified as physically active if they answered affirmatively to two of the three questions (21).

Medical information, encompassing multimorbidity and chronic conditions, was obtained by inquiring whether participants had received a medical diagnosis of hypertension, type 2 diabetes mellitus, chronic obstructive pulmonary disease, cardiovascular disease (including heart attack and angina), stroke, cancer, arthritis, osteoporosis, or sensory impairments like vision and hearing loss. Race and ethnicity were self-reported and classified into several categories: indigenous (encompassing various groups such as Ika, Kankuamo, Emberá, Misak, Nasa, Wayuu, Awuá, and Mokane); black, “mulatto,” or Afro-Colombian; white; and other (which includes mestizo, gypsy, etc.). Socioeconomic status was evaluated on a scale from one to six based on the housing stratum, with one denoting the highest level of poverty and six indicating the greatest wealth. This classification system, established by the National Government of Colombia, takes into account the physical attributes of the dwellings and their surrounding environments. The allocation into one of the six strata serves to reflect the hierarchical socioeconomic disparities ranging from poverty to wealth.

### 2.3 Diagnosis

The cut-off point of BMI was 30.0 kg/m^2^ for obesity, according to World Health Organization recommendations (14). Probable sarcopenia was defined as low HGS (22). Objective measures of muscle mass were not available, and calf circumference in the context of obesity can be significantly biased (23). Therefore, probable sarcopenia was identified using the EWGSOP2 sex-specific HGS cut-off points: <27 kg for men and <16 kg for women (24). Based on obesity status and HGS classification, participants were categorized into four phenotypes: Group A: non-obese with normal HGS; Group B: obese with normal HGS; Group C: non-obese with low HGS; Group D: obese with low HGS.

### 2.4 Statistical analysis plan

The baseline characteristics of the study participants are reported in terms of frequencies and percentages or as means and standard deviations (SD). Pearson chi-square tests were conducted to assess the statistically significant differences in the detection rates of cognitive impairment and probable dementia based on phenotype and sex. Binary logistic regression was executed to estimate the probabilities (Odds Ratio (OR)) for cognitive impairment and probable dementia by phenotype as independent variable. Models were progressively adjusted through Models 1, 2, and 3 with addition of covariates at each level, based on previously published literature (21). The present analysis was conducted without applying survey weights or accounting for the complex sampling design. All statistical analyses were performed using JASP (25). Statistical significance was established at *p* < 0.05.

## 3 Results

### 3.1 Characteristics according to sex

Table 1 shows the details of the people in the study, split by sex. Out of all, 57.5% were women with an average age of 69.7 years (SD 7.4), while 42.5% were men, averaging 70.3 years (SD 7.5). Looking at age groups, 51.9% of men and 56.3% of women were in the 60–69 range. Most participants were from a lower socio-economic class. Men drank alcohol and smoked more often than women. A lower proportion of women than men met the physical activity criterion used in this study (14.7% vs. 23.4%, respectively). Differences in phenotypes were seen. Among men, 43.4% had neither obesity nor low HGS, 10.6% were obese, 40.2% had low HGS, and 5.7% had obesity with low HGS. For women, 19.2% were obese, 35.1% had low HGS, and 15.1% showed obesity with low HGS. About 12.2% of men and 14.2% of women had cognitive impairment. Also, 4.9% of men and 6.6% of women were found to have probable dementia.

**Table 1:** Demographic characteristics of the participants by sex (*n* = 4,656)

Characteristics	Men	Women (2677)
	*n* = 1,979 (42.5%)	*n* = 2,677 (57.5%)
Age, mean (SD)	70.3 (7.5)	69.7 (7.4)
Age group, *n* (%)		
60–69	1,028 (51.9)	1,508 (56.3)
70–79	678 (34.3)	846 (31.6)
80+	273 (13.8)	323 (12.1)
Ethnic group, *n* (%)		
Indigenous	155 (7.8)	115 (4.3)
Black	185 (9.3)	200 (7.5)
White	508 (25.7)	768 (28.7)
Others	889 (44.9)	1,214 (45.3)
Socioeconomic status, *n* (%)		
Level I-II (low)	1,563 (79.0)	1992 (74.4)
Level III-IV (medium)	406 (20.5)	656 (24.5)
Level V-VI (high)	10 (0.5)	29 (1.1)
Residence area, *n* (%)		
Urban	1,458 (73.7)	2,142 (80.0)
Rural	521 (26.3)	535 (20.0)
Lifestyle outcomes, *n* (%)		
Alcohol consumption	467 (23.6)	133 (5.0)
Smoking	298 (15.1)	192 (7.2)
Meeting physical activity recommendations	463 (23.4)	394 (14.7)
Comorbid chronic diseases, *n* (%)		
HBP	941 (47.5)	1,627 (60.8)
Cholesterol	841 (42.5)	1,464 (54.7)
Diabetes	281 (14.2)	485 (18.1)
Cancer	86 (4.3)	141 (5.3)
COPD	194 (9.8)	290 (10.8)
CVD	261 (13.2)	392 (14.6)
Stroke	88 (4.4)	100 (3.7)
Arthritis	319 (16.1)	963 (36.0)
Osteoporosis	91 (4.6)	458 (17.1)
Phenotypes, *n* (%)		
Healthy	859 (43.4)	821 (30.6)
Obesity	211 (10.6)	514 (19.2)
Low Handgrip Strength	796 (40.2)	939 (35.1)
Obesity with low handgrip strength	113 (5.7)	403 (15.1)
Cognitive status, *n* (%)		
Non impairment	1,737 (87.7)	2,297 (85.8)
Impairment	242 (12.2)	380 (14.2)
Probable dementia status, *n* (%)		
Non impairment	1,776 (95.1)	2,382 (93.4)
Impairment	91 (4.9)	169 (6.6)

Values are presented as mean (SD) for continuous variables and numbers (percentages) for categorical variables. HBP, high blood pressure; COPD, chronic obstructive pulmonary disease; CVD, cardiovascular disease.

### 3.2 Cognitive impairment and probable dementia according to phenotypes by sex

Table 2 shows the frequency of cognitive impairment and probable dementia according to phenotype. The highest rates of cognitive impairment and probable dementia were found within the categories of low HGS and obesity with low HGS in both sexes. Among men, the frequency was very similar between the low HGS and obesity with low HGS phenotypes for both cognitive impairment (19.6% vs. 18.6%) and probable dementia (8.0% vs. 7.1%). Among women, the frequency was higher in the low HGS group than in the obesity with low HGS group for cognitive impairment (21.9% vs. 15.9%) and probable dementia (10.6% vs. 6.9%). In addition, among men, both outcomes were more frequent in the healthy phenotype than in the obesity phenotype. Statistically significant differences were observed across phenotypes in the distribution of cognitive impairment and probable dementia.

**Table 2:** Distribution of cognitive impairment and probable dementia according to phenotypes by sex.

Phenotypes, *n* (%)	Men		Women	
	Cognitive impairment	Probable dementia	Cognitive impairment	Probable dementia
Healthy	58 (6.8)	18 (2.1)	63 (7.7)	23 (2.8)
Obesity	7 (3.3)	1 (0.5)	47 (9.1)	18 (3.5)
Low handgrip strength	156 (19.6)	64 (8.0)	206 (21.9)	100 (10.6)
Obesity with low handgrip strength	21 (18.6)	8 (7.1)	64 (15.9)	28 (6.9)

238 missing in probable dementia category. All pairwise chi-squared comparisons between phenotypes were statistically significant (*p* < 0.05).

### 3.3 Regression analysis: odds of cognitive impairment and probable dementia by phenotype and sex

Table 3, displays the estimation odds of cognitive impairment or probable dementia according to phenotypes. Unadjusted OR (Model 1) analysis showed that low HGS is the condition more severe to develop both, cognitive impairment (OR 3.366; CI 2.447–4.630) in men and (OR 3.381; CI 2.505–4.564) in women, and probable dementia (OR 4.182; CI 2.455–7.126) in men and (OR 4.162; CI 2.617–6.619) in women (*p*-value <0.001). When adjusted the regressions by age and lifestyle habits (Model 2) the probabilities of cognitive impairment in men are incremental as we change phenotypes, i.e., low HGS (OR 1.859; CI 1.315–2.628) and obesity with low HGS (OR 2.022; CI 1.136–3.601). Likewise, in women, although slightly, the results show a greater probability of suffering cognitive impairment for low HGS (OR 2.085; CI 1.509–2.879) than for obesity with low HGS (OR 1.812; CI 1.227–2.676).

**Table 3:** Sex-stratified regression models for cognitive impairment and probable dementia

Outcome	Phenotype	MEN				WOMEN			
Model		Odds ratio	Lower 95% CI	Upper 95% CI	*p*-value	Odds ratio	Lower 95% CI	Upper 95% CI	*p*-value
Cognitive impairment									
Model 1	Non obesity/non sarcopenia								
	Obesity	0.471	0.213	1.058	0.068	1.214	0.814	1.797	0.347
	Low HGS	3.366	2.447	4.630	<0.001	3.381	2.505	4.564	<0.001
	Obesity with low HGS	3.152	1.830	5.430	<0.001	2.271	1.568	3.291	<0.001
Model 2	Non obesity/non sarcopenia								
	Obesity	0.566	0.252	1.272	0.168	1.421	0.945	2.139	0.092
	Low HGS	1.859	1.315	2.628	<0.001	2.085	1.509	2.879	<0.001
	Obesity with low HGS	2.022	1.136	3.601	0.017	1.812	1.227	2.676	0.003
Model 3	Non obesity/non sarcopenia								
	Obesity	0.547	0.241	1.240	0.148	1.501	0.980	2.298	0.062
	Low HGS	1.899	1.334	2.703	<0.001	2.317	1.654	3.246	<0.001
	Obesity with low HGS	2.090	1.155	3.781	0.015	2.142	1.416	3.239	<0.001
Probable dementia									
Model 1	Non obesity/non sarcopenia								
	Obesity	0.216	0.029	1.627	0.137	1.249	0.667	2.338	0.488
	Low HGS	4.182	2.455	7.126	<0.001	4.162	2.617	6.619	<0.001
	Obesity with low HGS	3.426	1.453	8.078	<0.001	2.655	1.507	4.675	<0.001
Model 2	Non obesity/non sarcopenia								
	Obesity	0.283	0.037	2.158	0.223	1.465	0.754	2.847	0.260
	Low HGS	1.678	0.937	3.004	0.081	1.962	1.183	3.254	<0.001
	Obesity with low HGS	1.816	0.731	4.512	0.199	1.878	1.028	3.432	0.040
Model 3	Non obesity/non sarcopenia								
	Obesity	0.265	0.034	2.049	0.203	1.676	0.834	3.367	0.147
	Low HGS	1.933	1.062	3.516	0.031	2.759	1.622	4.693	<0.001
	Obesity with low HGS	1.850	0.685	4.998	0.225	2.961	1.548	5.662	<0.001

HGS, handgrip strength; Model 1, unadjusted; Model 2, adjusted by age, tobacco, alcohol intake and physical activity (PA); Model 3, adjusted for the covariates included in Model 2 plus socioeconomic status, ethnic group, residence area, and comorbid chronic diseases.

Co-variables for cognition Model 2 Men: PA OR. 0.356 CI 95% (0.219–0.579); age OR. 1.111 CI 95% (1.089–1.134). Model 3 Men: PA OR. 0.382 CI 95% (0.233–0.625); age OR. 1.117 CI 95% (1.094–1.141). Model 2 Women: PA OR. 0.756 CI 95% (0.512–1.116); age OR. 1.111 CI 95% (1.094–1.129). Model 3 Women: PA OR. 0.935 CI 95% (0.624–1.402); age OR. 1.123 CI 95% (1.104–1.142).

Co-variables for probable dementia Model 2 Men: PA OR. 0.281 CI 95% (0.111–0.710); age OR. 1.155 CI 95% (1.119–1.193). Model 3 Men: PA OR. 0.293 CI 95% (0.114–0.753); age OR. 1.181 CI 95% (1.139–1.224). Model 2 Women: PA OR. 0.626 CI 95% (0.327–1.200); age OR. 1.157 CI 95% (1.130–1.184). Model 3 Women: PA OR. 0.828 CI 95% (0.421–1.627); age OR. 1.195 CI 95% (1.163–1.229).

**Figure 1:**
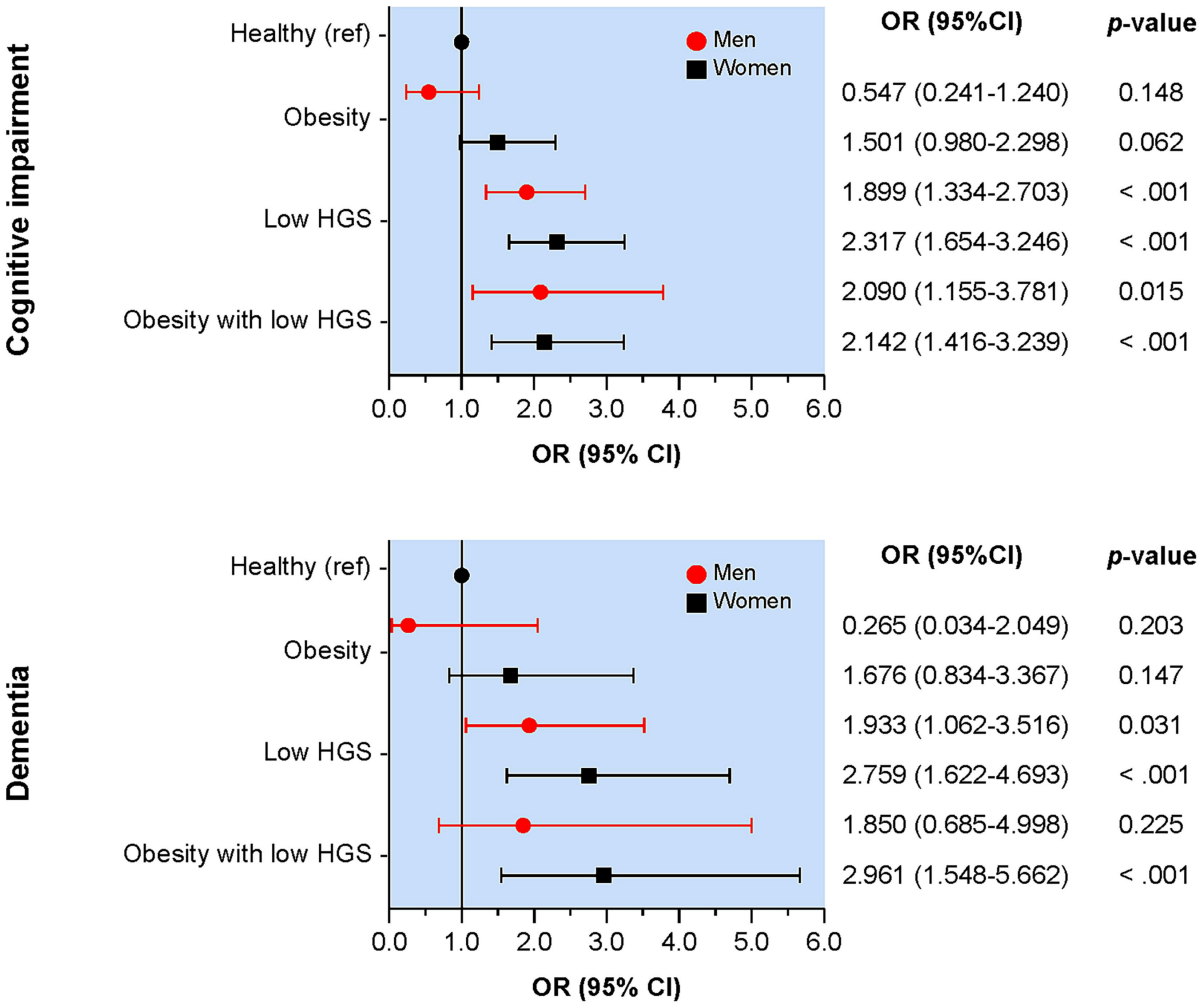
Forest plot of fully adjusted variables in regression analysis model for cognitive impairment and probable dementia by sex. Note: Model 3, fully adjusted by age, tobacco, alcohol intake, physical activity (PA), socioeconomic status, ethnic group, residence area and comorbid chronic diseases. Co-variables for cognition: Model 3 Men: PA OR. 0.382 CI 95% (0.233–0.625); age OR. 1.117 CI 95% (1.094–1.141). Model 3 Women: PA OR. 0.935 CI 95% (0.624–1.402); age OR. 1.123 CI 95% (1.104–1.142). Co-variables for probable dementia: Model 3 Men: PA OR. 0.293 CI 95% (0.114–0.753); age OR. 1.181 CI 95% (1.139–1.224). Model 3 Women: PA OR. 0.828 CI 95% (0.421–1.627); age OR. 1.195 CI 95% (1.163–1.229).

However, in men, the fully adjusted model showed that only low HGS was significantly associated with probable dementia (OR 1.933; 95% CI 1.062–3.516; *p* = 0.031), Figure 1. In women, the pattern was similar to that observed for cognitive impairment, with progressively higher odds from obesity to low HGS and obesity with low HGS. Physical activity was inversely associated with cognitive impairment and probable dementia in men, suggesting a lower likelihood of these outcomes among physically active participants.

## 4 Discussion

In this cross-sectional analysis of older adults, we show that low HGS alone, as well as obesity combined with low HGS, was associated with cognitive impairment and probable dementia. In contrast, obesity with a normal HGS was unrelated to these outcomes. These findings support the relevance of considering muscular status when examining cognitive health in older adults with obesity (8, 25). Recognizing and addressing sarcopenia or its components such as muscle strength can therefore guide interventions aimed at approaching a more holistic view of the individual.

Traditionally, BMI has been the most practical and widely used measure to assess nutritional status, cardiovascular and overall prognosis in the general population, largely because higher BMI values have been consistently linked to elevated risks of all-cause mortality and other adverse outcomes (13). However, recent and consistent evidence suggests this relationship changes with age: while the link between BMI and mortality appears stronger among younger individuals, older adults tend to have a higher “optimal” BMI associated with the lowest mortality risk, and the same trend has been reported with function and cognition (13, 26). Therefore, other variables related with body composition have taken hold, this is the case of those associated with the muscle (27, 28).

Age-related muscle wasting and dementia are major contributors to disability in older adults worldwide (29). Among adults aged 60 and above, prevalence estimates for sarcopenia range from about 5 to 13% in community-dwelling individuals. For those older than 70, prevalence may be higher, ranging from roughly 11 to 50% (30). Although primarily associated with aging, sarcopenia is exacerbated by factors such as physical inactivity, comorbidities, and inflammation (31). Sarcopenia has a range of detrimental clinical consequences that significantly affect overall health and quality of life. These include an increased risk of falls, which can result in serious injuries, fractures, and permanent dependency (31, 32). Moreover, sarcopenia contributes to a higher likelihood of disability, as muscle weakness impairs the ability to perform daily activities. Perhaps most concerning is its association with increased mortality rates (28). Recent literature suggests an association between muscular status and brain function, specifically cognitive decline (33). Sarcopenia is more prevalent in individuals with Alzheimer’s disease (AD), with estimates around 23.3% in AD and 12.5% in amnestic mild cognitive impairment (34). Furthermore, low muscle function may serve as an early marker for cognitive decline reinforcing this potential link (35).

Particularly, the most important attribute of muscle health is its function. Multiple studies have shown that lower grip strength is associated with an increased risk of cognitive decline and dementia, regardless of the dementia subtype (36). Even at middle age, reduced grip strength predicts a higher risk of all-cause dementia onset and mortality, independently of key confounding factors (37, 38). Probable sarcopenia is the presence of low muscle strength, it has been developed as intended to highlight muscle weakness as a key component of the whole sarcopenia concept (8). In a recent study in adults older than 75, it was shown that individuals with low HGS and obesity had significantly more cognitive decline over time than those low HGS or obesity (39).

In our study, we found that in men, higher levels of physical activity were linked to a lower risk of cognitive impairment and probable dementia in the regression models. Specifically, the odds ratios showed that being physically active cut the likelihood of these conditions by more than half. These findings reinforce the potential role of physical activity against cognitive impairment, and they align with systematic review studies demonstrating that regular physical activity lowers the incidence of cognitive impairment (40). These results were seen in men but not in women, presumably, as we could see in the frequency table because men met a higher percentage of the minimum required physical activity than women, hence the effect of reducing the probabilities.

This study has several limitations. First, because it is cross-sectional, we can only speculate about causation rather than establish it. Second, we performed a secondary analysis, indicating that the data were not initially collected for the specific aims of this research. Third, our reliance on self-reported comorbidities and demographic characteristics introduces the possibility of recall bias. Fourth, we did not measure muscle mass to confirm sarcopenia; however, our main goal was to highlight HGS as a useful independent variable. Fifth, although SABE Colombia 2015 employed a multistage probability sampling design with defined strata and primary sampling units, survey weights were not applied in the present analysis, which may affect the precision of confidence intervals and limits the generalisability of prevalence estimates to the broader Colombian older adult population. Sixth, BMI cannot distinguish fat mass from lean mass and may misclassify obesity in older adults subject to age-related body composition changes; direct measures such as DXA were unavailable in SABE Colombia 2015. Seventh, the ascertainment of probable dementia was not based on clinically confirmed diagnostic criteria applied by a physician; instead, we employed a population-based proxy widely used in Latin American epidemiological surveys (20) which may result in some degree of outcome misclassification. Although the study was conducted using a large population-based sample of community-dwelling older adults in Colombia, the generalizability of the findings to other populations may be limited. Lastly, our definition of low muscle mass does not alter the central findings, the prevalence of probable sarcopenia depends on the criteria used to define low handgrip strength, a threshold considered sufficient to prompt further assessment and intervention for sarcopenia (8).

Nonetheless, the study has several strengths. It is based on a large population-based dataset from a Latin American country, where evidence on these issues remains limited and where obesity, sarcopenia and dementia are highly prevalent. The anthropometric measurements were taken with validated and calibrated instruments. In addition, our results show how a simple, cheap and accessible method such is HGS can be an alternative to assess the presence other highly disabling conditions.

## 5 Conclusion

Our findings indicate that HGS, whether alone or in combination with obesity, is cross-sectionally associated with cognitive impairment and probable dementia in Colombian community-dwelling older adults, whereas obesity with normal HGS was not independently associated with these outcomes. These results support the relevance of considering muscular function when examining cognitive health in older adults with obesity. Prospective studies are needed to determine whether HGS may contribute to the identification or monitoring of individuals at greater risk of adverse cognitive outcomes.

## Data Availability

The Ministry of Health of Colombia manages the SABE Colombia dataset, which is publicly available. All respondent identifier information has been removed. Requests to access the dataset can be made by emailing repositorio@minsalud.gov.co.

## Footnotes

1
https://www.minsalud.gov.co/
