# Supplementary material for: The joint association of obesity and low handgrip strength with cognitive impairment and probable dementia: a cross-sectional study
Source: Front Nutr. 2026 Jul 17;13:1813754. doi: 10.3389/fnut.2026.1813754 (PMC13426277; doi:10.3389/fnut.2026.1813754)
Supplement: Supplementary file 1 [file Image_1.PDF]

Supplementary Material

eFigure 1. Flow diagram of participant selection for the analytic sample from the SABE Colombia 2015 cohort

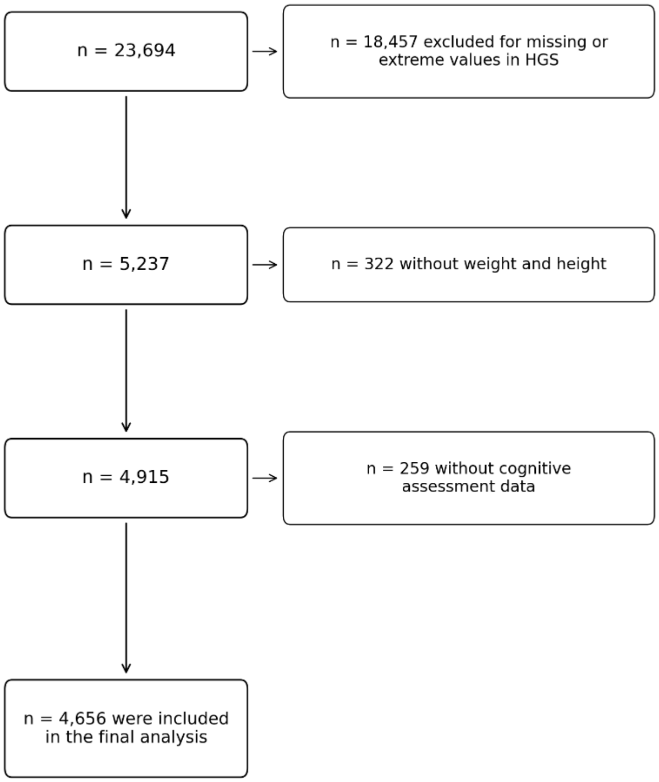

Footnote: HGS, handgrip strength. Of the 23,694 participants initially assessed, 18,457 were excluded due to missing or extreme HGS values, 322 due to missing anthropometric data (weight or height), and 259 due to missing cognitive assessment data, yielding a final analytic sample of 4,656 participants.
